# Understanding Type II Endoleak: A Harmless Imaging Finding or a Silent Threat?

**DOI:** 10.3390/jcm13144250

**Published:** 2024-07-20

**Authors:** Georgios Koudounas, Stefanos Giannopoulos, Nektarios Charisis, Nicos Labropoulos

**Affiliations:** 1 Vascular Unit, 5th Department of Surgery, Aristotle University Medical School, Hippokratio Hospital, 54642 Thessaloniki, Greece; georgekoudnas@gmail.com; 2 Division of Vascular and Endovascular Surgery, Department of Surgery, Stony Brook University Hospital, Stony Brook, NY 11794, USA; stefanos.giannopoulos@stonybrookmedicine.edu; 3 Department of Radiology, Stony Brook University Hospital, Stony Brook, NY 11794, USA; nektarios.charisis@stonybrookmedicine.edu

**Keywords:** type II endoleak, post-EVAR complication, transarterial embolization, sac embolization, aneurysmal sac expansion

## Abstract

Type II endoleak (T2EL) represents a challenging clinical entity following endovascular abdominal aortic aneurysm repair (EVAR). Although several studies have suggested that T2ELs are related to an increased risk of aneurysm sac growth and subsequent rupture, the exact role that T2ELs play in long-term outcomes remains debatable. Understanding the pathophysiology, diagnostic modalities, and management options of T2ELs is important for patients’ safety and proper resource utilization. While conservative management may be suitable for asymptomatic patients with a stable aneurysm size, interventional approaches, including transarterial embolization, direct sac puncture embolization and open conversion have been described for patients with persistent T2EL associated with sac expansion. However, more research is needed to better determine the clinical benefit of such interventions. A thorough evaluation of all endoleak types before T2EL treatment would be reasonable for patients with T2ELs associated with sac expansion. Further studies are needed to refine treatment strategies aimed at minimizing T2EL-related complications. Collaborative efforts among vascular specialists, radiologists, and researchers are of paramount importance to address this ongoing clinical challenge.

## 1. Introduction

Endovascular repair has revolutionized the management of abdominal aortic aneurysms (AAAs), offering a less invasive alternative to traditional open surgical techniques. Despite its many advantages, including reduced perioperative morbidity and mortality, endovascular aneurysm repair (EVAR) may lead to vexing complications and increase the reintervention rate [1,2]. One of the most common and clinically significant complications is the occurrence of endoleaks, which can result in persistent blood flow within the aneurysm sac and potential complications such as expansion and rupture [3].

Among the various types of endoleaks, type II endoleaks (T2ELs) stand out as particularly challenging due to their complex pathophysiology and variable clinical course. T2ELs are due to retrograde flow from patent aortic branches (i.e., lumbar, inferior mesenteric and accessory renal arteries) that perfuse the aneurysm sac. Once diagnosed, the management of T2ELs remains a topic of debate and controversy. 

While some patients may experience a spontaneous resolution of the endoleak over time, others may require intervention to prevent aneurysm sac expansion or rupture, especially if surveillance computed tomography (CT) shows an increasing aortic aneurysm size [4]. Various treatment modalities are available, including embolization of the aneurysm sac and branches or even open ligation of the feeding vessels. The selection of treatment depends on multiple factors, including the anatomical location of the endoleak, accessibility of the target vessels, and the patient’s overall health status [5].

We aim to provide a comprehensive review of T2ELs, explore the underlying pathophysiology, discuss the various diagnostic modalities available, and review the current evidence regarding management strategies and clinical outcomes. By synthesizing existing knowledge and highlighting areas of ongoing research and debate, we hope to contribute to a better understanding of this complex clinical entity and guide clinicians to the optimal management of patients presenting with T2ELs. 

## 2. Pathophysiology

T2ELs are the most common type of endoleaks, accounting for 50% of all endoleaks, and arise from persistence or reconstituted perigraft blood flow within the aneurysm sac following EVAR. The most frequent mechanism involves retrograde flow into the aneurysm sac through patent branches, such as lumbar arteries, inferior mesenteric artery (IMA), median sacral artery, or accessory renal arteries [6]. Regarding post-EVAR perioperative outcomes, T2EL is common at the time of implantation, present in 10–20% of patients at 1-month follow-up on CT scan [7,8,9]. T2ELs that remain patent after 6 months are considered persistent, reaching an incidence of 5–15% [9,10,11].

Several anatomical characteristics of aneurysms have been proposed as predisposing factors for persistent or late-developing T2ELs. More specifically, the number of lumbar arteries (more than 3) and their diameter (>2 mm) have been associated with a higher risk of developing a persistent T2EL [12,13,14]. A patent IMA with a diameter > 3 mm predisposes one to persistent T2EL as well [15,16]. On the other hand, preoperatively, an occluded IMA, an occluded L3 lumbar artery, and an occluded L4 lumbar artery are independently associated with protection against T2EL after EVAR and higher rates of sac regression [16]. 

Other characteristics, such as the proportion of aortic circumference lined with aortic thrombus, the maximum thrombus thickness, a longer aneurysm neck length, and ongoing anticoagulation have also been described as affecting the rate of T2ELs [17,18,19]. Demographics including older age, female sex, absence of chronic obstructive pulmonary disease (COPD), and hypogastric artery coil embolization might also serve as risk factors of persistent T2EL [9,18,20]. Lastly, whether T2EL is endograft-dependent or not remains a topic of debate, since some studies confirm an ongoing association, while others fail to detect it [9,21]. 

Approximately 30–75% of T2ELs appear to seal spontaneously, while the rest of them may persist in preventing complete aneurysmal sac thrombosis [5,22]. Interestingly, the timing of endoleak onset may alter the aneurysm sac behavior. A single-center retrospective study demonstrated that early T2ELs, defined as those identified within the first year postoperatively, self-resolved more frequently than late T2ELs, defined as onset >1 year following EVAR (75% vs. 29%) [23]. In the same study, among patients presenting with T2EL, the incidence of early- and late-occurring T2ELs was found to be 68% and 32%, respectively. Late T2EL patients are also more likely to suffer sac enlargement and subsequently necessitate treatment compared to patients with early T2EL. In plain words, although no clear pathophysiologic mechanism has been identified, late- vs. early-occurring T2ELs have been associated with higher rates of sac growth, likely attributed to continuous sac pressurization and expansion [23,24]. Alternatively, intra-aneurysm sac pressures to the level of the systemic blood pressure might lead to T2EL development, even with an angiographic absence of any endoleak post-EVAR graft deployment. 

Aortic sac enlargement can be observed in up to one fourth of patients with T2EL [4], and often occult type I and type III are revealed at the time of intervention, especially in cases with rapid sac growth [25]. Due to the enlargement of the aneurysm diameter caused by the persistence of T2EL, there is a risk of type IA or IB endoleak occurring as the proximal or distal landing zone shortens. Therefore, caution is required. Indeed, it is believed that progressive enlargement of the aneurysm sac may compromise the proximal or distal sealing zones, leading to high rates of reinterventions and conversion to open repair [26,27], although it is hard to prove whether these changes are solely attributed to T2EL or just reflect disease progression with further aortic degeneration over time. Nonetheless, as shown by a systematic review by Sidloff et al., the incidence of T2EL-associated aneurysm rupture is very low (0.9%) [28], and, thus, the overall clinical course of T2EL seems rather benign. On the other hand, type I and III endoleaks expose the aneurysm to direct aortic pressure, have the highest rupture risk (7.5% at 2 years and 8.9% at 1 year, respectively) and, therefore, necessitate prompt intervention [29]. Additionally, type IV endoleak and endotension are generally benign, may seal spontaneously, and usually do not require treatment. 

It is important to note that T2ELs may behave as an unpredictable indicator of worse outcomes, including multiple reinterventions, higher morbidity, and increased utilization of healthcare resources. Therefore, further research with large-scale real-world data is necessary to further investigate the natural course of T2EL and identify populations at risk. As technology is evolving and new-generation devices become commercially available, a decreasing rate of T2EL may be observed. 

## 3. Diagnostic Modalities and Post-EVAR Surveillance 

Different imaging modalities can be utilized to detect T2ELs during EVAR follow-up, including computed tomography angiography (CTA), magnetic resonance angiography (MRA), duplex ultrasonography (DUS), contrast-enhanced ultrasound (CEUS), and digital subtraction angiography (DSA). CTA is often the initial imaging modality of choice due to its widespread availability, high spatial resolution, and ability to provide detailed anatomical information [30]. Additionally, DUS is considered a valuable adjunct, augmenting CT scanning in EVAR surveillance, especially in cases where radiation exposure needs to be minimized.

More specifically, with regards to the identification of endoleaks, CTA achieves values of specificity and sensitivity of around 92% and 90%, respectively, exceeding those of classical DSA [31]. However, the downsides of CTA include difficulties in detecting very small endoleaks, the incorrect classification of endoleaks as type II instead of type I, an increased radiation exposure with a risk of malignancy, the administration of nephrotoxic contrasts, and high costs [32,33]. The use of non-contrast CT is limited and should be combined with DUS if the challenging diagnosis of T2EL is to be achieved. In light of this, four-dimensional (4D) CT is also a valuable adjunctive tool with a high sensitivity and specificity in endoleaks characterization. Four-dimensional CT and time-enhancement curve analysis, especially, may be superior to traditional biphasic CT protocols in revealing small endoleaks, predicting aneurysm sac growth, and determining the need for intervention in patients with T2EL [34,35]. 

DUS is a non-invasive, easily accessible, reproducible, and accepted alternative to CTA for the follow-up of EVAR patients with a high sensitivity in identifying endoleaks [36]. Despite being examiner- and patient-dependent, DUS eliminates the risk of ionizing radiation exposure and nephrotoxic contrast administration. It also permits low-cost repeated examinations, increasing its accuracy in T2EL detection. CEUS in particular shows sensitivity and specificity values similar to CTA, offering a real-time evaluation of T2ELs [37]. Nevertheless, echogenic contrast agents are not without contraindications, which include severe pulmonary hypertension, unstable angina, and a history of acute coronary syndrome. Finally, when combined with three-dimensional technology (3D-CEUS), it may be more accurate than even CTA in detecting and determining the type of endoleak [38].

MRA is also considered an alternative to CTA with similar outcomes. Habets et al. compared MRA to CTA regarding endoleak detection and concluded that MRA was more sensitive than CTA, in particular, for the identification of T2ELs [39]. Another systematic review highlighted the superiority of both DUS and MRA over CTA for the diagnosis of T2ELs, but this advantage seems to be lost when it comes to detecting type I and III endoleaks [40]. Therefore, MRA might play a role as an imaging modality for EVAR patients with aneurysm sac expansion and inconclusive CTA findings.

Although long-term regular follow-up post-EVAR seems meaningful due to persistent T2ELs and their potential association with adverse events, a large retrospective study did not reveal any survival benefit for patients with continued imaging follow-up vs. those without [41]. Interestingly, despite the establishment of standardized follow-up protocols for EVAR patients in the US and Europe, a recent meta-analysis highlighted that a significant number of T2ELs may be missed during the scheduled post-EVAR follow-up office appointments, even in cases where both CTA and CEUS were performed [42]. Therefore, further research is necessary to identify the causes for this observation and determine whether artificial intelligence could play a key role in this in the future, facilitating early detection of T2ELs. 

## 4. Management Strategies and Clinical Outcomes

The opposing outcomes of several well-designed studies have created controversy with regards to the management of T2ELs. Prevention and treatment of T2EL remain a topic of debate, and, currently, there is no consensus regarding the optimal approach. Most branch embolization studies focused on the IMA, aiming to reduce the likelihood of T2EL development before EVAR. Table 1 summarizes contemporary studies reporting on the outcomes of preemptive aortic branch embolization [43,44,45,46,47,48,49,50,51]. Aortic side branch embolization before EVAR was associated with a decreased incidence of T2EL (34.3% vs. 49.4%; *p* = 0.015) [45], fewer reinterventions (0.9% vs. 7.6%; *p* = 0.013) [45], and in several cases a greater aneurysmal sac shrinkage (5.2 mm vs. 2.1 mm) [46] compared to the non-embolization group. 

Other endovascular treatment options include transarterial embolization (TAE) and direct sac puncture embolization (DSPE), while transgraft and perigraft embolization have also been described [52,53,54,55,56]. DSPE can be performed percutaneously via left translumbar, transperitoneal, and right transcaval approaches. Studies investigating the outcomes of preemptive aneurysm sac embolization are displayed in Table 2 [57,58,59,60,61,62]. In the majority of the cases, sac embolization during EVAR resulted in a lower T2EL rate compared to patients undergoing standard EVAR (14.3% vs. 40.5%; *p* = 0.018) [57]. The choice of intervention depends on various factors, including the aneurysm morphology, location of the endoleak, accessibility of the target vessels, patient’s overall health status, and familiarity of the operator with the procedure.

The Society for Vascular Surgery (SVS) and European Society for Vascular Surgery (ESVS) guidelines recommend treatment of T2EL for aneurysm sac enlargement > 5 mm and 10 mm, respectively [63,64], although the quality of evidence is low. Clinical decision-making regarding treatment of T2EL is further challenged by the results of the ODYSSEOUS trial, a multicenter retrospective study of about 1600 EVAR patients that did not detect any difference in the overall survival in individuals with vs. without T2EL (45.9% vs. 44.1%; *p* = 0.54) [4]. Additionally, the study showed that reintervention for T2ELs did not achieve any survival advantage over conservative management [4]. Therefore, a more conservative approach with serial imaging may be a reasonable option for asymptomatic patients with a stable aneurysm size or slow disease progression [6,22]. Symptomatic patients and those with evidence of rapid aneurysm sac expansion may benefit from intervention, although multiple secondary interventions are often necessary in order to achieve sac stabilization [28,65,66]. 

Last, surgical treatment options with open side branch ligation or less invasive techniques, such as laparoscopic clipping of the IMA or the lumbars [67,68], have been described when embolization techniques fail to stabilize sac enlargement, and they are usually reserved only for select cases [69]. There are several open-surgery strategies for T2EL treatment, including total endograft explantation, partial endograft explantation, and complete endograft preservation. Since total stent graft explantation is more invasive, partial graft removal or even endoaneurysmorraphy with graft preservation are also valuable options aiming to avoid a suprarenal level of cross-clamping and extensive dissection, while reducing hemodynamic changes [70,71]. 

## 5. Prevention of T2EL and Future Developments

T2ELs are generally a benign clinical entity, with a low rate of life-threatening complications [28]. For this reason, no general guidelines have been released regarding their prevention. Many of the preemptive embolization studies revealed decreased rates of T2ELs post-EVAR and reinterventions, with higher rates of spontaneous resolutions [43,57,58,60]. This significantly decreased rate of endoleaks was found to be sustained even after 12 or 24 months in some studies [45]. The decreased rates of T2ELs along with the increased sac shrinkage rate could lead to a significant reduction of surveillance CT scans. Thus, the elimination of surveillance imaging examinations and secondary interventions may lead to the retrenchment of healthcare costs. 

Vaillant et al. compared the total cost of prevention to the cost of the readmission and subsequent reintervention to treat a persistent T2EL with sac enlargement and concluded that preoperative IMA embolization is a cost-effective technique [72]. The cost of preemptive embolization certainly varies among different states or countries. However, as shown by an Italian study, the cost of sac embolization with fibrin glue and coils is €1500, while the total cost for reintervention including the readmission can reach €9000 [73]. Undoubtedly, the universal appliance of preemptive embolization increases the immediate perioperative cost, procedure time, and likely the risk of complications such as kidney injury and radiation exposure. However, in patients with known risk factors, including ongoing anticoagulation therapy, an IMA > 3 mm or >3 patent lumbar arteries embolization prior to EVAR may be beneficial and cost-effective. Nonetheless, taking into consideration that the reintervention rate of T2EL after a standard EVAR is low, prospective studies evaluating the cost-effectiveness of the prevention modalities are warranted before the routine utilization of these techniques.

Additionally, as multiple opposing articles have been published leading to an unclear understanding of T2EL clinical significance, a list of essential questions about the T2EL natural course that need to be investigated in future research efforts is proposed in Table 3. 

## 6. Conclusions

The clinical significance of T2EL remains controversial, and its management continues to present a dilemma to clinicians. Serial imaging constitutes a reasonable option for asymptomatic patients with a stable aneurysm size or slow disease progression, while intervention could be reserved for cases of rapid sac growth. The true fate of T2EL and its effect on aneurysm sac remodeling remains unpredictable, with no direct link to aneurysm rupture and aneurysm-related mortality. A thorough evaluation of type IA, IB, and/or III endoleaks before T2EL treatment is reasonable in cases of sac expansion. Further studies are needed to better describe the natural course of T2EL and its association with baseline demographics and lesion characteristics, but also to investigate the benefit of preemptive branch and/or aneurysmal sac embolization before EVAR to prevent T2EL development. 

## Figures and Tables

**Table 1:** Studies investigating the outcomes of preoperative aortic branch embolization.

Study, Year	Population	Results & Conclusions
Samura et al., 2020 [51]	53 pts with IMA embolization during EVAR vs. 53 pts without	IMA embolization had lower T2EL incidence compared to the non-embolization group (13/53, 24.5% vs. 26/53, 49.1%; *p* = 0.009) and greater aneurysmal sac shrinkage (−5.7 ± 7.3 mm vs. −2.8 ± 6.6 mm; *p* = 0.037).
Manunga et al., 2017 [43]	258 pts with an attempted IMA embolization before EVAR vs. control group	Embolization protected against T2EL and led to fewer reinterventions.
Burbelko et al., 2014 [44]	Embolization with AMPLATZER plugs in 45 visceral and lumbar arteries with diameter > 2.5 mm	No T2EL postoperatively resulting in sac shrinkage in the embolization group.
Ward et al., 2013 [45]	108 pts with IMA embolization before EVAR vs. 158 pts with a patent IMA	IMA coil embolization prior to EVAR had reduced rate of T2EL compared to the non-embolization group (37/108, 34.3% vs. 78/158, 49.4%; *p* = 0.015), fewer secondary interventions (0.9% vs. 7.6%; *p* = 0.013), and fewer cases of increase in aneurysmal sac size at 24 months.
Nevala et al., 2010 [47]	40 pts with IMA coil embolization prior to EVAR vs. 39 pts without	Embolization led to a significantly lower T2EL rate (25% vs. 59%) but did not have any influence on the sac size.
Axelrod et al., 2004 [46]	102 pts with an attempted IMA embolization vs. control group	The non-embolization group had a significantly higher rate of T2EL, while the embolization group had greater shrinkage of the aneurysmal sac.
Bonvini et al., 2003 [48]	23 pts with preprocedural embolization of patent lumbar and IMA	There was only 1 (4.5%) T2EL from a patent lumbar artery, with no sac expansion after 2 years.
Parry et al., 2002 [49]	Preoperative successful IMA embolization in 13 of 16 pts and successful lumbar embolization in 8 of 13 pts	No T2ELs were developed in patients who underwent preoperative embolization, and in these cases a 3 mm median decrease in sac diameter was observed.
Gould et al., 2001 [50]	20 pts with successful or partly successful lumbar and IMA preoperative embolization vs. 43 pts without	20% T2EL rate and 0.5 mm mean sac shrinkage in the embolization group.

**Table 2:** Studies reporting on the outcomes of preemptive sac embolization.

Study, Year	Population	Results & Conclusions
Fabre et al., 2021 [57]	47 pts with aneurysm sac coil embolization during EVAR vs. 47 pts with standard EVAR	The embolization group had a significantly lower rate of T2EL at 12 months compared to pts with standard EVAR (14.3% vs. 40.5%). Nevertheless, this protection advantage was lost at 24 months.
Piazza et al., 2016 [58]	52 pts with intraoperative sac embolization vs. 55 pts with standard EVAR	The embolization group achieved higher freedom from T2EL at 3, 6, and 12 months, superior freedom from T2EL-related reintervention and greater shrinkage of the aneurysmal sac.
Zanchetta et al., 2007 [59]	84 pts with intraoperative intrasac fibrin glue injection	Sac embolization resulted in a low rate of delayed T2EL (2.4%) and a statistically significant decrease in the maximum transverse aneurysm diameter.
Mascoli et al., 2016 [60]	26 pts with intraprocedural sac embolization vs. 44 pts without	Selective intraoperative sac embolization in patients with known morphological risk factors decreases T2EL rate.
Muthu et al., 2007 [61]	69 pts with contemporary IMA embolization and thrombin injection into the sac vs. 69 controls	Despite the rate of T2EL being lower in the embolization group, no statistically significant results were achieved, mainly due to endoleaks from the lumbar arteries.
Pilon et al., 2010 [62]	18 pts with fibrin glue injection into the sac vs. 20 pts with standard EVAR	Sac embolization led to fewer CT scans, resulting in reduced health-care costs.

Abbreviations: pts, patients; EVAR, endovascular aneurysm repair; T2EL, type II endoleak; IMA, inferior mesenteric artery; CT, computed tomography.

**Table 3:** Suggested list of essential questions regarding T2EL fate.

Questions That Need to Be Discussed
Does T2EL affect survival?
Are late T2EL cases being missed?
Can an aneurysmal sac shrink despite T2EL?
How often should the surveillance be performed for T2ELs without sac expansion?
Which patients should have surveillance and for how long?
Does the timing of T2EL development affect aneurysm remodeling?
When do early T2EL cases start to experience sac shrinking?
How often should the assessment for type I or type III endoleaks be performed when an early T2EL is detected?
How often should the assessment for type I or type III endoleaks be performed when a late T2EL is detected?

Abbreviations: T2EL, type II endoleak.
